# RB1 dual role in proliferation and apoptosis: Cell fate control and implications for cancer therapy

**DOI:** 10.18632/oncotarget.4286

**Published:** 2015-06-18

**Authors:** Paola Indovina, Francesca Pentimalli, Nadia Casini, Immacolata Vocca, Antonio Giordano

**Affiliations:** ^1^ Sbarro Institute for Cancer Research and Molecular Medicine, Center for Biotechnology, College of Science and Technology, Temple University, Philadelphia, PA, USA; ^2^ Department of Medicine, Surgery and Neuroscience, University of Siena and Istituto Toscano Tumori (ITT), Siena, Italy; ^3^ Oncology Research Center of Mercogliano (CROM), Istituto Nazionale Tumori “Fodazione G. Pascale” – IRCCS, Naples, Italy

**Keywords:** RB family, apoptosis, E2F, cancer therapy, CDK inhibitors

## Abstract

Inactivation of the retinoblastoma (RB1) tumor suppressor is one of the most frequent and early recognized molecular hallmarks of cancer. RB1, although mainly studied for its role in the regulation of cell cycle, emerged as a key regulator of many biological processes. Among these, RB1 has been implicated in the regulation of apoptosis, the alteration of which underlies both cancer development and resistance to therapy. RB1 role in apoptosis, however, is still controversial because, depending on the context, the apoptotic cues, and its own status, RB1 can act either by inhibiting or promoting apoptosis. Moreover, the mechanisms whereby RB1 controls both proliferation and apoptosis in a coordinated manner are only now beginning to be unraveled. Here, by reviewing the main studies assessing the effect of RB1 status and modulation on these processes, we provide an overview of the possible underlying molecular mechanisms whereby RB1, and its family members, dictate cell fate in various contexts. We also describe the current antitumoral strategies aimed at the use of RB1 as predictive, prognostic and therapeutic target in cancer. A thorough understanding of RB1 function in controlling cell fate determination is crucial for a successful translation of RB1 status assessment in the clinical setting.

## INTRODUCTION

Inactivation of the retinoblastoma (RB1) tumor suppressor occurs with such a high frequency in cancer — either directly, through mutations, or indirectly, through the altered expression of RB1 regulators — to be proposed as a fundamental event for tumor development [1]. Therefore, many efforts have been dedicated to investigate RB1 mechanisms of action in order to shed light on key events in cancer development.

Early studies identified RB1 and other cell cycle regulatory proteins as intracellular targets of transforming viruses [2–5]. So, initial studies on RB1 function focused on its role as a cell cycle regulator and correlated its oncosuppressive activity mainly with its capability to negatively regulate cell cycle through the interaction with members of the E2F family of transcription factors [6]. Indeed, the canonical model for the tumor suppressive action of RB1, which emerged from these early studies, is based on its ability to inhibit the G1-S transition through the repression of E2F target genes involved in DNA synthesis and cell cycle progression. This function of RB1 is regulated through changes in its phosphorylation status, which are mediated by cyclin-cyclin-dependent kinase (CDK) complexes, CDK inhibitors and phosphatase activity (for a review see [7]). When RB1 is in its active hypophosphorylated state, it represses E2F-mediated transcription by binding and blocking the E2F transactivation domain and forming complexes with E2Fs, together with their heterodimerization partners DPs, at cell cycle gene promoters. Conversely, RB1 phosphorylation, which is initiated by cyclin D-CDK4/6 in response to mitogenic signals, inactivates the RB1 repressive function by dissociating the RB1-E2F-DP complexes. The transcriptional repressive activity of RB1 also relies on its ability to recruit chromatin remodelling enzymes to E2F-regulated promoters. However, the action of these enzymes seems to be more related to chromatin changes associated with a stable gene silencing during cell cycle exit rather than to the negative control of the G1-S transition in cycling cells [8].

RB1 belongs to a family of three proteins, including also retinoblastoma-like 1 (RBL1/p107) and retinoblastoma-like 2 (RBL2/p130) [7]. RBL2/p130 altered expression and delocalization was found in various cancers, in which it functions as a valuable prognostic marker [9–21]. Both RBL1/p107 and RBL2/p130 are involved in the negative regulation of cell cycle by interacting with E2F family members although in different combinations with respect to RB1 [7]. Recently, it has been shown that RBL2/p130, together with E2F4-DP and the multi-vulval class B (MuvB) core component, form the DREAM complex (DP, RB-like, E2F and MuvB), which mediates cell cycle gene repression during quiescence and coordinates periodic gene expression during the G1-S and G2-M transitions by releasing RBL2/p130 and recruiting different transcription factors [22].

However, although classically the tumor suppressive function of the RB proteins has been mainly attributed to their ability to arrest cell cycle by repressing E2F target genes, these proteins can also control cell cycle progression through E2F-independent mechanisms. Indeed, both RB1 and RB-like proteins are able to negatively regulate cell cycle by inhibiting CDK activity through either indirect or direct mechanisms, respectively [8, 23]. Moreover, beyond their function as G1 checkpoint regulators, RB proteins are involved in many other cellular processes, such as preservation of chromosomal stability, induction and maintenance of senescence, regulation of cellular differentiation, angiogenesis, and apoptosis, which could all contribute to the RB oncosuppressive activity [24, 25]. At the molecular level, RB1 is now viewed as a platform for multiple protein interactions through which it regulates different cellular pathways [8].

Given the central role of RB1 in regulating cellular processes that are crucial for both defense against tumor progression and response to cancer treatments, a careful analysis of RB1 status could be critical to guide therapeutic decisions. Furthermore, unleashing RB1 oncosuppressive potential is an appealing strategy for cancer therapy. Considering that the main goal of most anticancer therapeutic interventions is to induce apoptotic cell death or inhibit proliferation, a fundamental requirement to exploit RB1 for therapeutic purposes is to fully understand its role in these processes. However, whereas the role of RB proteins as negative regulators of cell proliferation is well-understood, their role in apoptosis is still very controversial. Here, we provide an overview of the main findings on the function of RB1 and the other family members in the apoptotic process and in the coordinated control of cell death and proliferation in both normal cells and preclinical cancer models. Moreover we describe clinical studies exploring the possible utility of RB1 as a predictive marker of response to both cytotoxic and cytostatic treatments and studies investigating the possible use of RB proteins as ultimate therapeutic targets.

### RB family and apoptosis regulation

The role of RB1 in apoptosis control is still very controversial and limited data are available for as concerns the role of the other RB family members in this process. The first observations indicated an antiapoptotic role of RB1. However, emerging data now show a proapoptotic role of RB1 in different cellular contexts and recent advances in the understanding of RB1 mechanisms of action in apoptosis suggest multifunctional roles for this protein, which can be modulated by several post-translational modifications [8].

#### Early findings revealing the antiapoptotic role of RB1

The ability to evade apoptosis is a hallmark of cancer [26]. Consistently, alterations that inhibit apoptosis can favor tumor progression and cause resistance to treatments [27]. So, it was quite surprising when findings from mouse studies first revealed an antiapoptotic role for RB1, considering its well-established oncosuppressive function. Indeed, studies with *Rb1*-null mice showed that RB1 loss induced not only deregulated proliferation, as expected, but also massive apoptosis in the nervous system, lens and skeletal muscles (see [28] for a thorough description of these studies).

Genetic crosses demonstrated that the RB1-binding partners E2Fs have a crucial role in triggering apoptosis in *Rb1*-null mice [28]. Indeed, as a consequence of RB1 loss, the release and de-repression of the E2F1 transcription factor, which among the E2F family members has a unique role in apoptosis induction [29], can trigger cell death by the activation of E2F1 target genes encoding proapoptotic proteins, such as p73, caspases, apoptotic peptidase activating factor-1 (APAF-1), and BCL-2 homology 3 (BH3)-only family members [30].

However, later it became clear that the apoptotic phenotypes of *Rb1*-null mice are not entirely caused by E2F deregulation but are also largely due to the defective development of extra-embryonic tissues rather than only to the lack of *Rb1* cell autonomous function [31–33]. Moreover, RB1 is able to bind and inhibit proapoptotic factors other than E2F1 [28].

The analysis of tissue-specific *Rb1* mutant mouse models showed that RB1 loss in some tissues induced unscheduled proliferation without having effects on apoptosis, whereas in other tissues (lens and myoblasts) induced apoptosis, specifically in differentiating cells [34]. It has been suggested that RB1 loss can induce either apoptosis or uncontrolled proliferation depending on different cellular contexts: in cells committed to a specific differentiation program RB1 deficiency triggers apoptosis, whereas in cycling cells RB1 loss leads to uncontrolled proliferation [35]. A possible explanation on how cells lacking RB1 can proliferate rather than undergo apoptosis is that mitogenic stimulation activates prosurvival factors that counteract the proapoptotic gene induction resulting from RB1 loss [28].

#### Role of RB1 in the coordinated control of proliferation and apoptosis

**Figure 1:**
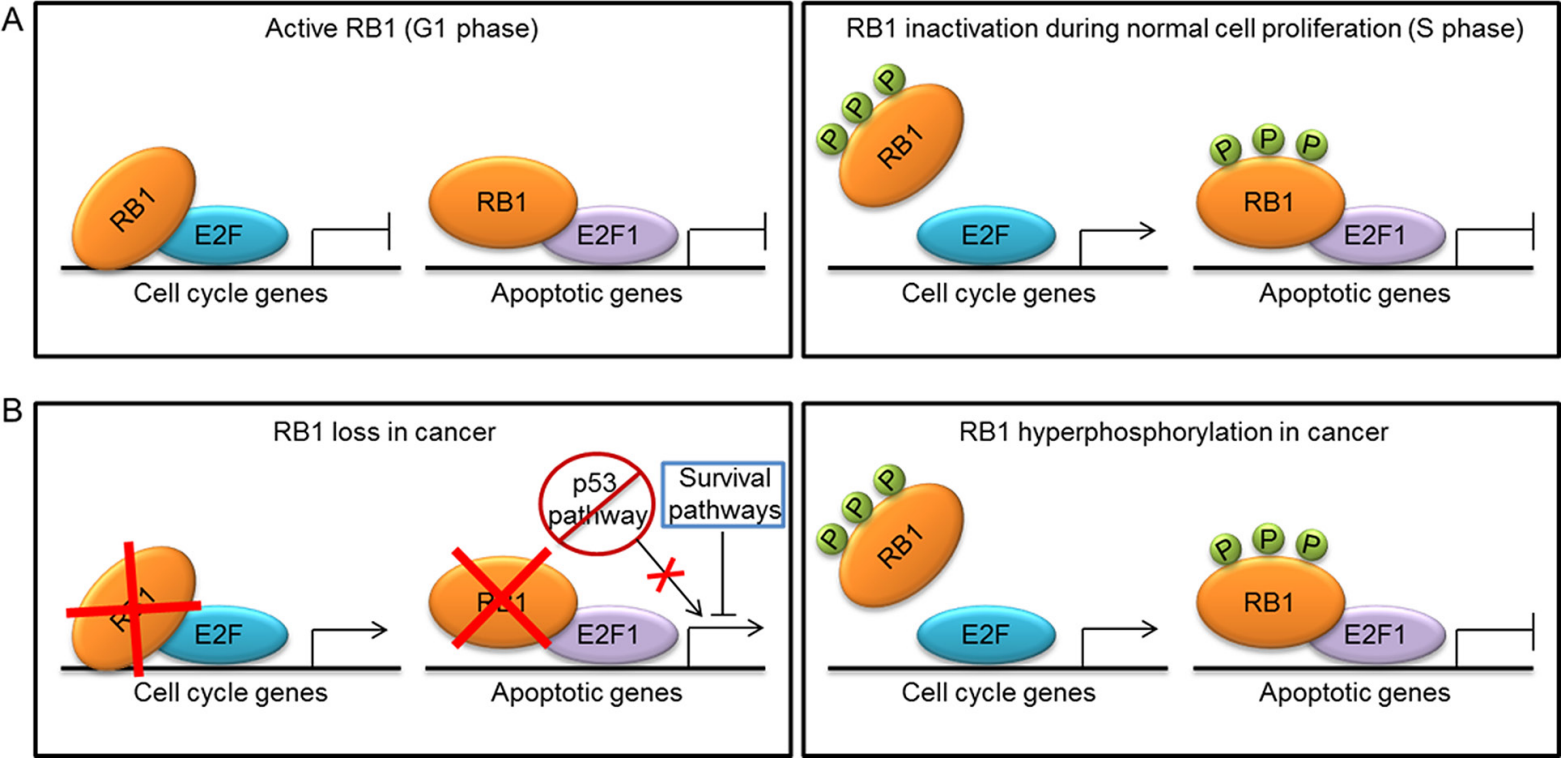
Mechanisms of RB1 inactivation during normal cell proliferation and tumorigenesis and their effect on transcription of cell cycle and apoptotic genes **A.** In G1 phase, the active hypophosphorylated form of RB1 binds to E2F transcription factors to repress the expression of cell cycle and apoptotic genes. In S phase, RB1 is inactivated by phosphorylation (P) and releases most E2Fs to induce transcription of cell cycle genes. A fraction of RB1-E2F1 complexes persist at the promoters of apoptotic genes, thus repressing their expression. **B.** In cancer, RB1 can be inactivated by either mutations or hyperphosphorylation. RB1 loss leads to de-repression of both cell cycle and apoptotic genes, whereas hyperphosphorylation causes de-repression only of cell cycle genes. Thus, in cells lacking RB1, tumorigenesis can occur only if survival pathways protect cells from RB1-loss induced apoptosis by limiting E2F1 proapoptotic activity or if a second alteration, such as the abrogation of the p53 proapoptotic pathway, occurs.

RB1 dual role as inhibitor of both cell division and apoptosis raises the question of how normal cells can inactivate RB1 to enable cell division without inducing apoptosis. A possible mechanistic explanation is that the RB1 reversible inhibition occurring during cell cycle through phosphorylation is functionally different from the RB1 complete loss that induces apoptosis in *Rb1*-null mice [36]. Support for this hypothesis comes from the observation that *Cdkn2a*-null animals, in which RB1 is expected to be in a hyperphosphorylated state, do not show the massive apoptosis observed in *Rb1*-null mice [36]. So, the “inactive” hyperphosphorylated form of RB1 seems to maintain the antiapoptotic activity. Indeed, although the commonly accepted model for RB1 function would predict that phosphorylated RB1 releases its E2F partners during G1-S cell cycle transition, it has been observed that RB1-E2F1 complexes persist beyond the S phase, regardless of RB1 hyperphosphorylation [37]. Moreover, in proliferating cells RB1 and E2F1 occupy apoptotic gene promoters, whereas are dissociated from cell cycle genes [38]. The ability of RB1-E2F1 complexes to persist when RB1 is phosphorylated during cell cycle progression seems to rely on an E2F1-specific binding domain in the RB1 C-terminal region [8]. Therefore, a simplified model that emerges from these observations is that phosphorylation of RB1 by CDKs during G1-S transition causes the release of most E2Fs to induce transcription of cell cycle genes, but at least a fraction of RB1-E2F1 complexes remains stable thanks to the unique interaction of these proteins and persist at the promoters of apoptotic genes, thus repressing their expression [8] (Fig. 1A).

#### RB1 antiapoptotic role and tumorigenesis

RB1 role in inhibiting apoptosis has important implications for tumorigenesis. RB1 ability to prevent apoptosis might seem inconsistent with its oncosuppressive role, since apoptosis inhibition is more likely to favor tumor growth. However, the induction of apoptosis, which occurs as a consequence of RB1 loss, can represent a safeguard mechanism to hinder the expansion of potentially malignant cells [39]. Indeed, RB1 inhibition confers a proliferative advantage to cells, which could favor tumor development, but the massive apoptosis resulting from RB1 loss could overcome the increased proliferative potential and hinder cancer growth. This can explain why most sporadic cancers have RB1 inactivated because of defects in the pathways that regulate its phosphorylation rather than by mutations [28]. In this way, tumors gain a proliferative advantage and, at the same time, can be protected from apoptosis by the hyperphosphorylated form of RB1, which could preserve its antiapoptotic activity through the same mechanism described above (Fig. 1B). Another proposed mechanism whereby phosphorylated RB1 can protect cells from apoptosis is by directly binding and inhibiting the proapoptotic factor ANP32A (acidic nuclear phosphoprotein 32A) [40]. Therefore, RB1 phosphorylation can provide cancer cells with both proliferative and survival advantages.

Conversely, following RB1 loss, cancer can develop only if cells are intrinsically resistant to RB1-deficiency-induced apoptosis, or if cell death is counteracted by parallel activation of survival pathways, or, finally, if a second alteration, such as the abrogation of the p53 proapoptotic pathway (Fig. 1B), occurs during tumor development to allow cell expansion and cancer growth [28]. A recently described mechanism of apoptosis inhibition whereby RB1-deficient cells can undergo malignant transformation involves the de-repression of the S-phase kinase-associated protein 2 (SKP2), which suppresses apoptosis by limiting E2F1 activity [41]. A further mechanism of resistance to apoptosis that could promote tumorigenesis following RB1 loss could rely on the increased levels of the antiapoptotic protein survivin. Indeed, it has been demonstrated that RB1 is able to inhibit transcription of the gene encoding survivin [42–44] and homozygous deletion of *Rb1* in mouse embryonic fibroblasts (MEFs) led to survivin induction [42]. Consistently, high levels of survivin were found in the *RB1*-null cancer retinoblastoma [45].

#### RB1 antiapoptotic role in response to cell death inducers

Several observations from *RB1* knockdown and overexpression studies confirmed the antiapoptotic role of RB1 also in response to different apoptotic stimuli. In particular, *RB1* knockdown has been shown to enhance the sensitivity to cell death induced by different anticancer agents, such as DNA-damaging and microtubule interfering agents, in cells from several cancer types, including lymphoma, breast, lung, and prostate cancer, and glioblastoma [46–50]. Similarly, RB1 ablation in mouse embryonic and adult fibroblasts increased the sensitivity to chemotherapy-induced cell death [51–53]. Analogously, restoration of the wild-type RB1 protein in RB1-deficient cells from several cancer types (osteosarcoma and different carcinomas) inhibited apoptosis upon various apoptotic stimuli, such as ionizing radiation, p53 overexpression, ceramide, and interferon (IFN)-γ [54–57]. Therefore, all these data point to a protective role of RB1 against different cell death inducers in several cell types. Some studies suggested that this protective action could be a secondary consequence of RB1 ability to arrest cell cycle in response to stress signals [52, 58, 59]. However, the ectopic expression of a mutated form of RB1, which was unable to induce growth arrest, protected RB1 deficient osteosarcoma and breast cancer cells from DNA damage-induced apoptosis [60]. Thus, RB1 can exert an antiapoptotic activity independent of growth suppression, probably mainly through the direct inhibition of apoptotic genes.

#### Role of RB1 dephosphorylation and caspase cleavage during apoptosis

Apoptosis is often accompanied by a shift from the hyperphosphorylated to the hypophosphorylated form of RB1 [61–67]. Consistently, phosphatase activity directed toward RB1 seems to be required for apoptosis induction in cells from different cancer types [61, 65, 67, 68] and the antiapoptotic protein BCL2 can prevent RB1 dephosphorylation and apoptosis [63, 64]. Moreover, RB1 hyperphosphorylation seems to be correlated with resistance to apoptotic treatments [69, 70]. All these studies suggest that RB1 dephosphorylation is required for apoptosis to occur, and, in particular, it has been recently reported that dephosphorylation at threonine-821 has a key role in this process [71].

Studies conducted on promyelocytic leukemia and breast cancer cell lines suggested that dephosphorylation of RB1 during apoptosis could be necessary for its cleavage by caspases and consequent degradation, which would eliminate its antiapoptotic action and allow cells to undergo death in response to apoptotic stimuli, such as DNA damage [65, 67, 72, 73]. Indeed, RB1 dephosphorylation seems to be a prerequisite for caspase cleavage, since the hyperphosphorylated form of RB1 is a poor caspase substrate [65], and different studies support the notion that caspase cleavage is a crucial step for eliminating RB1 during apoptosis in various cell types [74–78].

**Figure 2:**
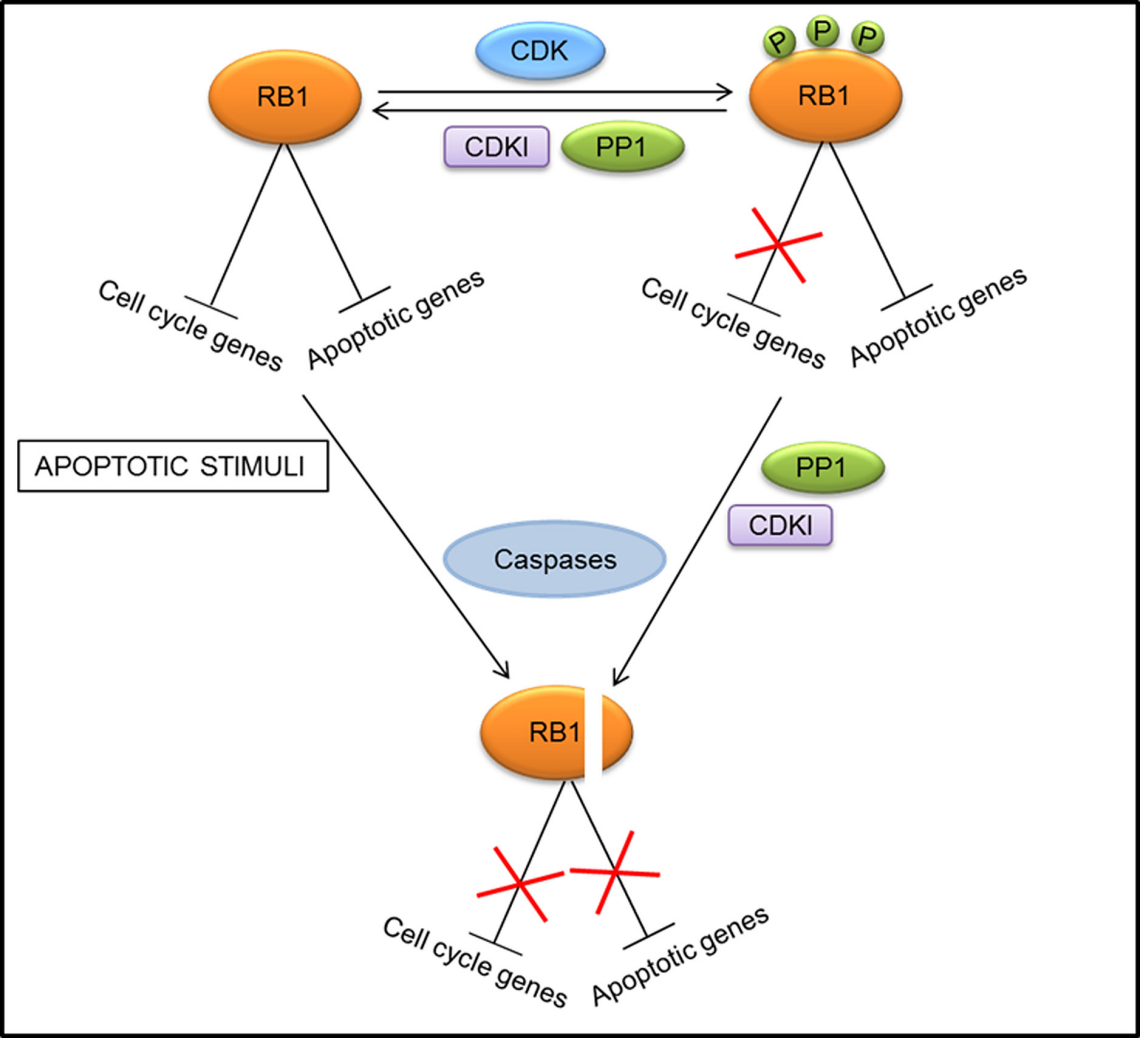
Different mechanisms of regulation of RB1 activity at the promoters of cell cycle and apoptotic genes At the promoters of cell cycle genes, RB1 activity is modulated through phosphorylation (P)/dephosphorylation processes mediated by cyclin dependent kinases (CDKs), CDK inhibitors (CDKIs), and protein phosphatase 1 (PP1). Conversely, phosphorylation by CDKs does not affect the RB1 repressive action at promoters of apoptotic genes. A mechanism to prevent this inhibitory activity and induce apoptotic gene expression consists in RB1 dephosphorylation and subsequent cleavage by caspases.

So, it was tempting to speculate that, unlike the promoters of cell cycle genes where RB1 activity is mostly modulated through phosphorylation/dephosphorylation processes, at the promoters of apoptotic genes RB1 degradation could play an important part [28] (Fig. 2). However, although caspase-resistant forms of RB1 hindered apoptosis induced by tumor-necrosis factor-alpha (TNF-α) in mouse fibroblasts [79, 80] and by potassium deprivation in mouse cerebellar neurons [81], the cleavage-resistant RB1 did not decrease cell death triggered by DNA damage in mouse fibroblasts [80] and did not protect human T lymphocytes from apoptosis induced by the ligation of FAS death receptor [79]. Thus, RB1 cleavage by caspases is not an absolute prerequisite for apoptosis, but it can be a crucial step for certain types of apoptotic events, depending on both the cell type and the nature of the death inducers.

#### Emerging data revealing a proapoptotic role for RB1

The RB1 dephosphorylation observed during apoptosis is not always linked to a subsequent RB1 degradation, which, as described above, could serve to eliminate its antiapoptotic action. Conversely, the active hypophosphorylated form of RB1 seems to have a crucial role in apoptosis induction in some circumstances. Indeed, accumulation of the active hypophosphorylated form of RB1 seemed to be required for apoptosis induced by matrix contact deprivation in prostate cancer cells [64]. Consistently, the induced expression of RB1 forms that cannot be phosphorylated and/or degraded by caspases stimulated the apoptotic response to TNF in Rat-16 cells [59]. Overall these data point to a proapoptotic role of RB1 in some contexts, although the phosphorylation resistant form of RB1 causes contrasting effects in response to different apoptotic stimuli [59].

Support for the hypothesis of a proapoptotic role of RB1 in some circumstances also comes from a study reporting that *RB1* knockdown reduced the toxicity of histone deacetylase (HDAC) inhibitors in colon cancer cells [82]. Moreover, the restoration of wild-type *RB1* in *RB1*-deficient prostate cancer cells promoted apoptosis after radiation or ceramide treatment [83, 84]. This is, however, in sharp contrast with the aforementioned studies showing apoptosis inhibition following the restoration of wild-type RB1 in several RB1-deficient cell types upon different treatments.

**Figure 3:**
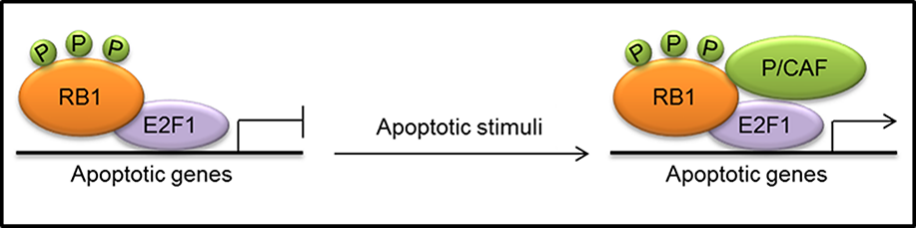
Formation of a transcriptionally active complex, consisting of phosphorylated (P) RB1, E2F1 and the histone acetyltransferase p300/CBP-associated factor (P/CAF), at the promoters of apoptotic genes in response to apoptotic stimuli

A molecular mechanism whereby RB1 can exert a proapoptotic function in some cellular contexts in response to apoptotic stimuli, such as DNA damage, has been recently proposed [8, 34]. In response to DNA damage, both RB1 and E2F1 undergo extensive post-translational modifications: RB1 is dephosphorylated at CDK target sites but is phosphorylated by checkpoint kinase 1/2 (CHEK1/2), acetylated, and methylated; E2F1 is phosphorylated by ataxia-telangiectasia mutated (ATM) and CHEK2, as well as acetylated and demethylated [8, 85]. Phosphorylated RB1 remains bound to E2F1 and the recruitment of the histone acetyltransferase p300/CBP-associated factor (P/CAF; also known as KAT2B) leads to the formation of a transcriptionally active complex, which promotes the expression of pro-apoptotic genes (Fig. 3). Consistently, a transcriptionally active complex containing RB1, E2F1 and P/CAF has also been reported to have a key role in the apoptotic response following hepatocarcinoma cell treatment with transforming growth factor-β1 (TGF-β1) [86].

Most of the findings on the role of RB1 in apoptosis derived from studies on its activity as transcriptional regulator. Conversely, in a recent study RB1 was shown able to promote apoptosis in response to apoptotic signals at mithocondria, by directly interacting with BAX and conformationally activating it to induce mitochondrial outer membrane permeabilization and cytochrome c release [30, 87]. Moreover, hyperphosphorylated RB1 is still competent to induce mitochondrial apoptosis. However, another study showed that RB1 dephosphorylation caused RB1-BAX complex dissociation and apoptosis induction [88]. These incongruous results could be explained by the different cell types used in the two studies (MEF and human breast cancer cell, respectively) or by the different methods used for manipulating phosphorylation, which might result in differences in site-specific phosphorylation of RB1 [88].

In conclusion, all these observations indicate that RB1 is a multifunctional protein, whose role in apoptosis depends on both the cell type and the nature of the death inducers and whose function can be modulated by different combinations of phosphorylated sites and other post-translational modifications.

#### Role of the other RB family members in apoptosis

Although data on the function of RBL1/p107 and RBL2/p130 in apoptosis are more limited than those available for RB1, the picture that emerges is similarly complex.

Studies with mutant mice suggested an antiapoptotic role for RBL1/p107 and RBL2/p130. Indeed, although *Rbl1*-null mice did not show increased cell death during embryogenesis, mouse embryos lacking both *Rb1* and *Rbl1* showed accelerated apoptosis in liver and central nervous system with respect to embryos lacking only *Rb1* [89]. Therefore, RBL1/p107 seems to cooperate with RB1 in protecting some cell types from apoptosis. Balb/cJ mouse embryos lacking *Rbl2* showed extensive apoptosis in the neural tube, brain and dermomyotome, thus suggesting an antiapoptotic role for RBL2/p130 at least in this mouse strain [90]. Moreover, although the individual loss of each *Rb* family member did not significantly affect proliferation and survival of mouse pancreatic β cells *in vivo*, the combined loss of *Rb1* and *Rbl2* resulted in increased proliferation and cell death [91].

Consistent with a possible antiapoptotic role of RBL2/p130, it has been observed that its overexpression inhibits apoptosis in response to apoptotic stimuli in neurons [92] and ovarian cancer cells [93].

At the molecular level, the RBL2/p130-E2F4 repressor complex has been shown to inhibit directly the transcription of apoptotic genes in postmitotic cardiomyocytes [94] and neurons [92]. Apoptotic stimuli can induce RBL2/p130 hyperphosphorylation, dissociation of the RBL2/p130-E2F4 complex and de-repression of apoptotic genes in neurons [92]. So, unlike the RB1 repressive activity at the apoptotic genes, which seems to be maintained regardless of RB1 phosphorylation (Fig. 1), the RBL2/p130 inhibitory action at these genes can be inactivated through RBL2/p130 phosphorylation. Consistently, the RB family members are inactivated through different mechanisms during camptothecin-induced apoptosis in B lymphocytes: RB1 is degraded, in keeping with the above described role of RB1 caspase cleavage during apoptosis in various cell types, whereas RBL1/p107 and RBL2/p130 are phosphorylated, leading to the release of E2F proteins [95]. Consistent with this different regulation of RB protein activity during apoptosis, the C-terminal caspase cleavage site of RB1 is not conserved in RBL1/p107 and RBL2/p130 [74].

Although these observations indicate an antiapoptotic role for RBL1/p107 and RBL2/p130, the latter can also exert a proapoptotic activity in different contexts. In particular, in marked contrast to what observed in mouse pancreatic β cells lacking both *Rb1* and *Rbl2* [91], the combined ablation of these two genes in the mouse lung epithelium resulted in reduced apoptosis with respect to that observed in lung epithelium lacking only *Rb1*, thus suggesting a proapoptotic activity of RBL2/p130 [96]. Consistently, the expression of RBL2/p130 was correlated with high apoptotic indexes in retinoblastoma samples [97]. Moreover, RBL2/p130 overexpression induced apoptosis in Saos-2 osteosarcoma cells [97] and marrow stromal cells committed toward a neuron-like phenotype [98] and caused an increase in γ-radiation-induced apoptosis in hamster glioblastoma cells [99].

Interestingly, RBL2/p130 can be part of different protein complexes with opposite functions at apoptotic gene promoters. Indeed, it has been suggested that the difference in apoptotic response to chemotherapeutic treatment observed in drug-resistant osteosarcoma cells and their parental cell line could be due to the presence of distinct E2Fs–RBL2/p130 complexes on the p73 promoter: in the parental cell line, p73 transcription is activated by an E2F1–RBL2/p130–p300 complex, whereas, in the drug-resistant cells, it is repressed by an E2F4–RBL2/p130–HDAC1 complex [100]. So, the involvement in functionally different complexes provides a possible molecular explanation on how RBL2/p130 can exert opposite functions in apoptosis regulation in different circumstances. However, we are still far from understanding the mechanisms whereby RBL2/p130 can contribute to apoptosis control and further research in this field is ongoing.

### RB1 as a predictive marker of response to therapy

The key dual role of RB1 in arresting cell cycle upon anti-proliferative signals and in apoptotic response to various anticancer treatments, observed in the preclinical studies described above, suggests that RB1 could be a crucial determinant of clinical response to therapies. Therefore, a careful evaluation of its functional status and context-dependent role in response to specific treatments in different tumors might be fundamental to guide therapeutic decisions. Indeed, several studies on patients with different cancer types suggest that RB1 status affects tumor sensitivity to treatments and clinical outcome (Table 1). Therefore, RB1 might represent a predictive marker of response to therapy for various tumor types.

**Table 1:** Studies on the role of RB1 as a predictive marker of response to therapies

Cancer type	Treatments	RB1 status and assessment method	No of patients	Association between RB1 status and clinical response	References
**ALL**	Glucocorticoids	Hypophosphorylation (HRE and WB)	32	RB1 hypophosphorylation correlates with good responsiveness to glucocorticoid therapy.	[115]
**Bladder cancer**	Radiation	Loss of expression (IHC)	98	Loss of RB1 expression is associated with improved response to radiation.	[110]
**Bladder cancer**	Radiation	Loss of expression (IHC)	106	Loss of RB1 expression is associated with improved response to radiation and relapse-free survival.	[111]
**Bladder cancer**	Transurethral resection and intravesical BCG +IFN-α	Low expression (IHC)	93	RB1 underexpression is associated with nonresponse to BCG +IFN-α treatment and tumor recurrence.	[113]
**Bladder cancer**	Transurethral resection and intravesical BCG	Loss of expression or overexpression (IHC)	27	RB1 altered expression predicts recurrence and progression following BCG treatment.	[114]
**Breast cancer**	Cyclophosphamide, methotrexate, 5-FU	Loss of expression (IHC)	518	Loss of RB1expression predicts a good clinical outcome for patients treated with adjuvant chemotherapy.	[101]
**Breast cancer**	Paclitaxel, 5-FU, doxorubicin, cyclophosphamide	LOH (analysis of polymorphic markers at RB1 locus)	133	RB1-LOH signature is associated with a good response to neoadjuvant chemotherapy.	[102]
**Breast cancer**	Cyclophosphamide, methotrexate, 5-FU	Loss of expression (IHC)	518	RB1 loss is higher in TNBCs than in other subtypes. In patients with TNBCs treated with adjuvant chemotherapy, RB1 loss is associated with a good prognosis.	[103]
**Breast cancer**	Three different neoadjuvant chemotherapy regimens	Pathway disruption (analysis of an RB1-loss gene expression signature)	98	RB1 pathway disruption is associated with improved response to multiple chemotherapeutic regimens in both ER+ and ER− breast cancers.	[104]
**Breast cancer**	Tamoxifen	Pathway disruption (analysis of an RB1-loss gene expression signature)	60	RB1 pathway deregulation is associated with early recurrence following tamoxifen monotherapy in ER+ tumors.	[47]
**Breast cancer**	Tamoxifen	Loss of function (discrepancy between RB1 phosphorylation and cell proliferation)	500	Loss of RB1 function is associated with tamoxifen treatment resistance in ER+ tumors.	[105]
**Ovarian carcinoma**	Platinum and paxlitaxel chemotherapy combinations after radical surgery	High expression (IHC)	300	High expression of RB1 is associated with a poor prognosis in patients who underwent radical surgery and postoperative chemotherapy.	[116]
**Prostate cancer**	Combined androgen blockade	Low mRNA levels (RT-PCR)	81 tumors	A higher frequency of *RB1* mRNA downregulation was observed in cancers from treated patients with respect to those from untreated patients.	[107]
**Prostate cancer**	Hormone deprivation	Loss (RT-qPCR, IHC, gene expression signature, locus copy number)	44	RB1 loss is associated with the transition to the incurable castration-resistant status and poor clinical outcome.	[108]

ALL: acute lymphoblastic leukemia; BCG: bacillus Calmette-Guerin; ER: estrogen receptor; 5-FU: 5-fluorouracil; HRE: high resolution electrophoresis; IFN-α: interferon-α; IHC: immunohistochemistry; LOH: loss of heterozygosity; RT-qPCR: reverse transcription-quntitative PCR; TNBC: triple-negative breast cancer; WB: western blotting

In particular, consistent with the above reported observation that *RB1* knockdown enhances the sensitivity to cell death induced by DNA-damaging agents in breast cancer cells [47], the disruption of RB1 function was found to be associated with improved response to adjuvant/neoadjuvant chemotherapy and good clinical outcome for patients with breast cancer [101–104]. Conversely, RB1 pathway deregulation in breast cancers proved to be associated with resistance to hormone therapy with the antiestrogen tamoxifen and tumor recurrence [47, 105]. These observations are consistent with preclinical data showing that RB1 knockdown or functional inactivation in breast cancer cells prevents antiestrogen-induced cell cycle arrest [47, 106], thus resulting in continued proliferation and tumor xenograft growth [47]. Overall, these studies indicate that the evaluation of *RB1* status is crucial for directing breast cancer therapy, suggesting, in particular, that patients with RB1-negative breast cancers might benefit from chemotherapeutic treatments, whereas disruption of RB1 function is associated with failure of hormone therapy.

Similar to breast cancer, also in prostate cancer RB1 deficiency seems to be associated with a poor response to hormone therapy (including surgical or chemical castration to suppress androgen production and/or administration of antiandrogens). Indeed, a higher frequency of *RB1* mRNA downregulation was observed in recurrent prostate cancers from patients who had undergone combined androgen blockade with respect to cancers from untreated patients, suggesting that RB1 inactivation might be associated with hormone treatment resistance [107]. Also, RB1 loss in prostate cancer samples was found to be associated with the transition to the incurable castration-resistant status and poor clinical outcome [108]. These results are consistent with preclinical data showing that RB1 knockdown or functional inactivation in prostate cancer cells inhibits the androgen deprivation-induced proliferative stall [49, 109] and is sufficient to induce castration-resistant tumor xenograft growth [108]. Conversely, as also reported above, RB1 knockdown enhanced the sensitivity of prostate cancer cell lines to cell death triggered by antimicrotubule and DNA-damaging agents [49]. Therefore, RB1 status seems to have a key role in cellular response to therapeutic interventions in prostate cancer cells and could represent a marker for directing therapy also in this tumor type.

Consistent with the putative antiapoptotic role of RB1 in bladder cancer cells, as observed following ceramide treatment in the aforementioned study by McConkey [56], and in keeping with the above described protective role of RB1 against DNA-damaging agents in several cancer types, loss of RB1 expression was found to be associated with improved response to radiation and relapse-free survival in patients with bladder cancer [110, 111]. Therefore, the analysis of RB1 status might be useful to select patients with bladder cancer who would benefit from a treatment schedule including radiotherapy, which has been shown to induce ceramide [112]. Conversely, RB1 altered expression seems to be predictive of nonresponse to intravesical bacille Calmette-Guerin (BCG) +/− IFN-α treatment and tumor recurrence in bladder cancer patients [113, 114].

Interestingly, RB1 hypophosphorylation occurring upon treatment with glucocorticoids, the main pharmacological agents used against childhood acute lymphoblastic leukemia (ALL), correlates with a good responsiveness [115], suggesting that RB1 might represent a possible surrogate endpoint of therapeutic response for childhood ALL.

Finally, high RB1 expression was correlated with a poor prognosis in ovarian carcinoma patients who had undergone radical surgery and postoperative chemotherapy [116] and also to be associated with vascular invasion and recurrence in hepatocellular carcinoma patients after surgical treatments [117]. These studies suggest that high RB1 expression holds the potential to be a good prognosticator of postoperative disease for these tumor types, and could be used to stratify patients for postoperative treatment in certain instances.

In addition to affect cellular sensitivity to cytotoxic and hormonal agents routinely used in the clinic, RB1 status also impacts cellular response to a number of kinase inhibitors, which, although not yet in clinical use, represent the basis for the development of promising targeted therapies. In particular, similar to what described for hormone therapy, RB1 deficiency seems to limit the effectiveness of these agents that function through cytostatic mechanisms [118].

Although the aforementioned studies point to an important role of RB1 as a predictor of therapeutic response, the evaluation of its status is not yet used to guide therapeutic decisions in clinical practice. Indeed, the major hurdle associated with the development of RB1 as a predictive marker of response to treatments is the lack of standardized methods to assess its functional status in tumor specimens [118] and several different approaches have been used to detect the different mechanisms whereby RB1 is functionally inactivated in cancer (Table 1).

### Targeting RB1 for cancer therapy

Owing to RB1 involvement in many cellular processes, all contributing to its oncosuppressive activity, and considering that its pathway is altered in most human cancers, RB1 represents an appealing target for cancer therapy. Two main strategies have been conceived to directly target RB1 for cancer treatment. The first aims to exploit RB1 loss for therapeutic purposes, whereas the second is intended to reactivate RB1 tumor suppressor function [119].

#### Exploiting RB1 deficiency to therapeutic purposes

**Figure 4:**
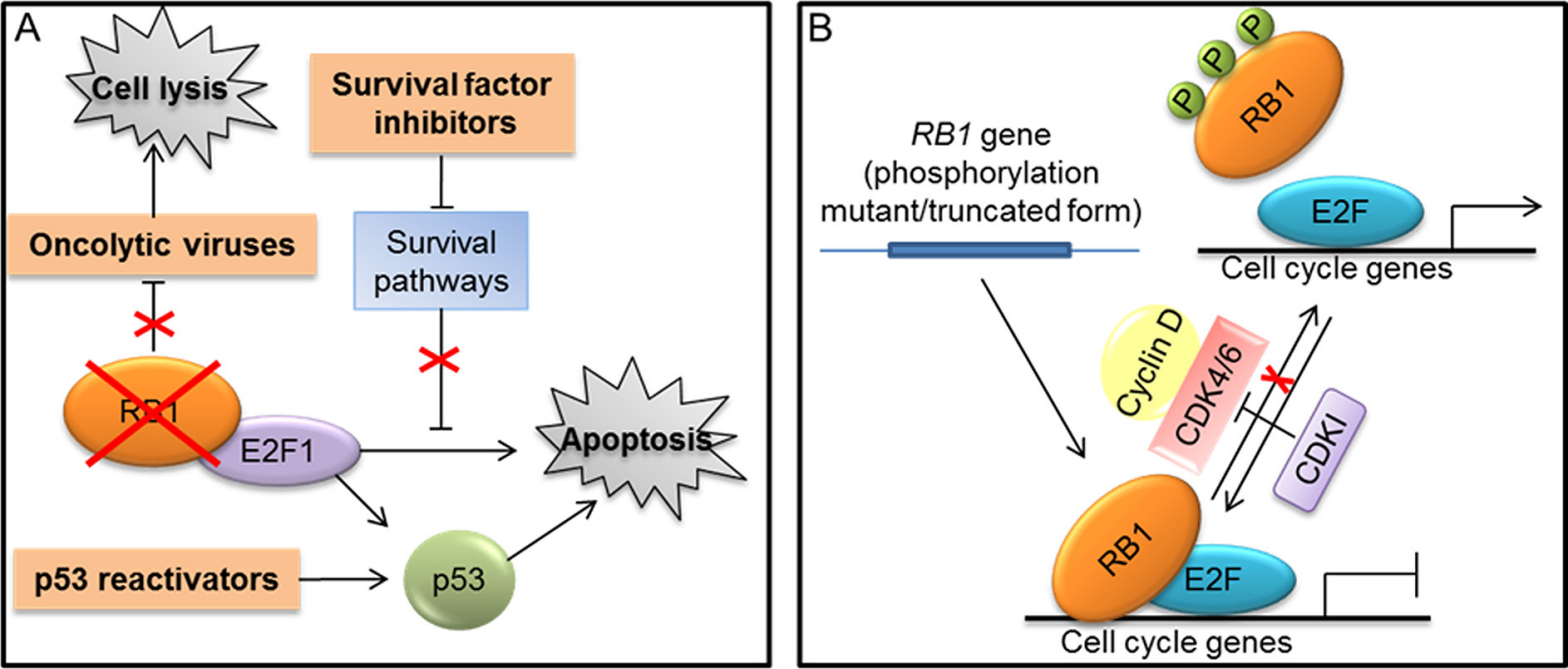
Strategies targeting RB1 for cancer treatment **A.** Approaches that exploit RB1 loss to therapeutic purposes (indicated in orange boxes): use of oncolytic viruses that depend on RB1 inactivation for their replication and tumor cell killing; methods that exploit the loss of RB1 and the consequent E2F1 activation to kill tumor cells through the use of agents, such as inhibitors of survival factors and p53 reactivators, that enhance the E2F-1 mediated apoptosis. **B.** Approaches aiming to reactivate RB1 tumor suppressor function: gene-transfer strategies to express truncated or mutated forms of RB1 that remain in the hypophosphorylated status; RB1 reactivation through the use of cyclin dependent kinases inhibitors (CDKIs) to maintain an efficient transcriptional repression and induce cell cycle arrest.

Different approaches exploiting RB1 loss to kill cancer cells have been designed (Fig. 4A). For instance, oncolytic viruses carrying deletions in the E1A viral protein, which render viral replication dependent on RB1 inactivation, were developed to selectively kill RB1-defective tumor cells [120] (Fig. 4A). Moreover, other oncolytic viruses were designed to exploit the deregulated E2F transcriptional activity resulting from RB1 loss for their replication and tumor cell killing [120]. Similarly, another approach took advantage of deregulated E2F transcriptional activity to activate a prodrug selectively in cancer cells with inactive RB1 [121]. Furthermore, chemical/genetics screen have been performed to identify compounds or “synthetic lethal” genes to specifically kill RB1-defective cells [122, 123].

Another strategy to selectively kill RB1-deficient cells is based on agents able to release the latent E2F1 proapoptotic activity in these cells [118, 119]. Indeed, as described above (Fig. 1B), in cancer cells lacking RB1, the E2F1-mediated apoptosis resulting from RB1 deficiency is suppressed through resistance mechanisms [28]. Different cellular pathways that protect cells from RB1-loss-induced apoptosis by limiting E2F1 proapoptotic activity have been identified [41, 118, 119]. Therefore, inhibitors of these survival pathways could be synthetically lethal with RB1 deficiency and could represent the basis to develop a therapeutic approach targeting RB1-defective cells [118, 119] (Fig. 4A).

The E2F1 proapoptotic activity is interconnected with that of p53 (see [124] for a thorough description of this molecular crosstalk) and, as illustrated above (Fig. 1B), the abrogation of p53 proapoptotic pathway could be required to limit apoptosis downstream of RB1 loss in order to enable tumorigenesis [28]. For instance, inhibition of p53 pathway is a crucial determinant in the genesis of the *RB1*-null cancer retinoblastoma [125]. Thus, it has been shown that compounds reactivating p53 could be useful in the treatment of retinoblastoma and other tumors lacking RB1, through the increase of E2F1-mediated apoptosis [118, 119, 126] (Fig. 4A).

#### Restoring RB1 tumor suppressor function

Compared to other tumor suppressors, such as p53, fewer studies aiming to restore RB1 function have been reported. Restoration of wild-type RB1 protein through gene-transfer approaches produced variable results on tumor cell growth and showed deleterious effects on p53-mediated apoptosis. Therefore, RB1 phosphorylation mutants and truncated variants that remain in the hypophosphorylated status have been used in preclinical models to enhance RB1 tumor suppressor function [120, 127] (Fig. 4B).

Another strategy to reactivate RB1 function is based on the use of CDK inhibitors that can prevent RB1 phosphorylation (Fig. 4B). However, whether these compounds exert their effects mainly acting on RB1 pathway remains an unanswered question, especially considering that most of the available molecules target multiple CDKs, including also transcription-related CDKs, can have also other targets, and seem to act also through mechanisms independent from cell cycle [128–132]. Nevertheless, a potent and highly selective inhibitor of CDK4 and CDK6, called Palbociclib (PD-0332991), recently proved to be dependent on the presence of RB1 for its antiproliferative effect in several cancer cell lines and xenograft models [133] and a Phase I trial of PD-0332991 utilized RB1-deficiency as an exclusion criterion [119]. In a Phase II trial, PD-0332991 treatment was associated with a favorable progression-free rate in patients with CDK4-amplified and RB-expressing liposarcomas [134]. Moreover, in patients with mantle cell lymphoma, the antiproliferative effect of PD-0332991 was significantly correlated with a marked decrease in the percentage of phospho-RB1 positive cells in lymph node biopsies [135]. The outcome of preclinical and clinical trials based on the use of PD-0332991 have been recently reviewed by Knudsen and colleagues [136].

Consistent with the above reported observations that RB1 pathway disruption is correlated with resistance to therapies that function through cytostatic mechanisms, such as hormone therapy, reactivation of the antiproliferative activity of RB1 through CDK4/6 inhibitors in combination with endocrine therapy have shown promise in breast cancer clinical trials [137]. However, considering that highly selective CDK4/6 inhibitors, such as PD-0332991, act only as cytostatic agents without inducing apoptosis [138–141], an important issue is to establish whether these compounds favor or antagonize apoptotic induction by the commonly used cytotoxic agents. It has been reported that G1 cell cycle arrest induced by pretreatment or concurrent treatment with highly selective CDK4/6 inhibitors prevented apoptotic triggering by different chemotherapeutics in RB1-positive breast cancer cells, melanoma cells and immortalized fibroblasts, thus suggesting that CDK4/6 inhibitors can antagonize chemotherapy [137, 139, 141]. Conversely, contrasting effects have been observed following combination treatments with CDK4/6 inhibitors and ionizing radiation. Indeed, pretreatment with highly selective CDK4/6 inhibitors (PD-0332991 or 2BrIC) increased radioresistance of RB1-positive melanoma cells and immortalized fibroblasts [139], whereas combination treatments with radiation administered either concurrently or sequentially with PD-0332991 resulted in increased anticancer activity in mice with RB1-positive glioblastoma intracranial xenografts [140]. Moreover, PD-0332991 sensitized myeloma cells to cell death induced by different cytotoxic agents, such as the glucocorticoid dexamethasone, the proteasome inhibitor bortezomib and the immunomodulatory agent lenalidomide, as indicated by both preclinical studies and clinical trials [138, 142–144]. Therefore, a thorough understanding of the effects of CDK4/6 inhibition in combination with different cytotoxic agents is required to define the best treatment schedule in a clinical setting. For instance, previous studies have shown that the “cell cycle-mediated resistance” to chemotherapy can be overcome by an appropriately scheduled administration [136, 145].

Data on the possible use of the other RB family members as targets in cancer therapy are very limited, although retrovirus-mediated transfer of RBL2/p130 proved to be able to inhibit lung carcinoma cell growth both *in vitro* and in a mouse model [11]. Moreover, we developed a 39-residue peptide, called Spa310, derived from the spacer region of RBL2/p130, which was able to inhibit CDK2 activity and lung cancer proliferation in xenotrasplanted nude mice, thus suggesting that this peptide could represent the basis for the development of new targeted therapeutic strategies [146–148].

## CONCLUSIONS

Since its discovery as the first tumor suppressor gene almost 30 years ago, *RB1* has represented an appealing target for cancer therapy. Indeed, decades of research have been dedicated to investigate the mechanisms of action of RB1 protein in order to exploit it therapeutically. In particular, considering that the main goal of most anticancer therapies is to inhibit proliferation and induce apoptosis, a major focus of this research has been to fully understand RB1 role in these processes both in physiological conditions and in response to treatments.

Unlike the well-established function of RB1 as a negative regulator of cell cycle, its role in apoptosis is still very controversial. Early studies with *Rb1*-null mice revealed an antiapoptotic role for RB1. Thus, reconciling the seemingly incongruous functions of RB1 as a negative regulator of both proliferation and apoptosis has represented a particularly tough challenge for many years. However, recent studies have identified molecular mechanisms, mainly based on the unique interaction between phosphorylated RB1 and the proapoptotic factor E2F1, able to explain RB1 function in the coordinated control of proliferation and apoptosis both in normal cells and during tumorigenesis.

RB1 has an antiapoptotic action also in response to different death stimuli and, at least in some apoptotic events, this action can be eliminated through RB1 dephosphorylation and subsequent caspase cleavage. However, to further complicate matters, emerging data have revealed that RB1 can exert a proapoptotic function in some cell types upon different treatments. Thus, RB1 is now viewed as a multifunctional protein, whose role in apoptosis depends on both the cell type and the nature of the death inducers and whose function can be modulated by several post-translational modifications. Therefore, a careful evaluation of its functional status and context-dependent role in response to specific treatments in different tumors might be fundamental to guide therapeutic decisions.

Several clinical studies suggest, indeed, that RB1 might represent a predictive marker of response to therapy for various tumor types. Overall, RB1 status seems to have opposite effects on responses to cytotoxic and cytostatic agents, respectively. In particular, RB1 deficiency seems to be associated with improved cytotoxic response to DNA damaging agents, at least for some cancer types. Conversely, the loss of RB1 function limits the effectiveness of anti-proliferative signals and hormone therapies, which function through cytostatic mechanisms.

Taken together, all these observations identify RB1 as crucial target of tailored anticancer strategies. Two main approaches have been conceived to exploit RB1 therapeutically. The first aims to take advantage of RB1 loss mainly through the research of synthetic lethal interactions and the use of oncolytic viruses, which depend on RB1 inactivation for their replication and tumor cell killing. Conversely, the second is intended to reactivate RB1 tumor suppressor function, principally through the use of CDK4/6 inhibitors that have shown promise in clinical trials.

Despite these promising results, to date a greater understanding of RB1 functions is required to successfully translate RB1-related research to the clinic. Indeed, the importance of RB1 functional status in therapeutic response has not yet been elucidated for many cancer types. Moreover, the development of more rigorous, standardized methods to assess RB1 status in tumor specimens is required before RB1 can be used as a predictive marker to guide therapeutic decisions in clinical practice. Furthermore, a deep understanding of the specific functions of different combinations of phosphorylations and other post-translational modifications is necessary to fully clarify the RB1 biological activity in different contexts and to define the best therapeutic strategy.

## Abbreviations

ALL: acute lymphoblastic leukemia
ANP32A: acidic nuclear phosphoprotein 32A
PAF-1: apoptotic peptidase activating factor-1
ATM: ataxia-telangiectasia mutated
BCG: bacillus Calmette-Guerin
BH3: BCL-2 homology 3
CDK: cyclin-dependent kinase
CDKI: CDK inhibitor
CHEK1/2: checkpoint kinase 1/2
DREAM: DP, RB like, E2F and MuvB
ER: estrogen receptor
5-FU: 5-fluorouracil
HDAC: histone deacetylase
HRE: high resolution electrophoresis
IFN-α: interferon-α
IHC: immunohistochemistry
LOH: loss of heterozygosity
MEFs: mouse embryonic fibroblasts
MuvB: multi-vulval class B
P/CAF: p300/CBP-associated factor
PP1: protein phosphatase 1
RB: retinoblastoma
RBL: retinoblastoma-like
RT-qPCR: reverse transcription-quntitative PCR
SKP2: S-phase kinase-associated protein 2
TGF-β1: transforming growth factor-β1
TNBC: triple-negative breast cancer
TNF-α: tumor-necrosis factor-alpha
WB: western blotting
